# Identification of co-expressed central genes and transcription factors in atherosclerosis-related intracranial aneurysm

**DOI:** 10.3389/fneur.2023.1055456

**Published:** 2023-03-02

**Authors:** Quan Zhang, Hengfang Liu, Min Zhang, Fang Liu, Tiantian Liu

**Affiliations:** ^1^Department of Neurology, Fifth Affiliated Hospital of Zhengzhou University, Zhengzhou, Henan, China; ^2^Department of Neurology, First Affiliated Hospital of Zhengzhou University, Zhengzhou, Henan, China

**Keywords:** intracranial aneurysms, atherosclerosis, bioinformatics, differentially expressed genes, hub genes, transcription factor

## Abstract

**Background:**

Numerous clinical studies have shown that atherosclerosis is one of the risk factors for intracranial aneurysms. Calcifications in the intracranial aneurysm walls are frequently correlated with atherosclerosis. However, the pathogenesis of atherosclerosis-related intracranial aneurysms remains unclear. This study aims to investigate this mechanism.

**Methods:**

The Gene Expression Omnibus (GEO) database was used to download the gene expression profiles for atherosclerosis (GSE100927) and intracranial aneurysms (GSE75436). Following the identification of the common differentially expressed genes (DEGs) of atherosclerosis and intracranial aneurysm, the network creation of protein interactions, functional annotation, the identification of hub genes, and co-expression analysis were conducted. Thereafter, we predicted the transcription factors (TF) of hub genes and verified their expressions.

**Results:**

A total of 270 common (62 downregulated and 208 upregulated) DEGs were identified for subsequent analysis. Functional analyses highlighted the significant role of phagocytosis, cytotoxicity, and T-cell receptor signaling pathways in this disease progression. Eight hub genes were identified and verified, namely, CCR5, FCGR3A, IL10RA, ITGAX, LCP2, PTPRC, TLR2, and TYROBP. Two TFs were also predicted and verified, which were IKZF1 and SPI1.

**Conclusion:**

Intracranial aneurysms are correlated with atherosclerosis. We identified several hub genes for atherosclerosis-related intracranial aneurysms and explored the underlying pathogenesis. These discoveries may provide new insights for future experiments and clinical practice.

## 1. Introduction

Intracranial aneurysm (IA) is a prevalent disease that affects ~3% of the population (1). The rupture of an IA leads to subarachnoid hemorrhage, with a high risk of morbidity and death. Although the pathogenesis of IA remains unclear, it may be closely related to atherosclerosis (AS). An increasing number of studies have found that IAs are frequently complicated by AS, resulting in a worse prognosis. Killer-Oberpfalzer et al. found atherosclerotic lesions in all their deaths from cystic IA (2). Evidence supports the hypothesis that atherosclerosis, inflammation, and degenerative changes in aneurysm walls play considerable roles in the development of IA, and the presence of atherosclerotic plaques in the aneurysm wall may contribute to the degeneration and rupture of IA (3, 4). Aneurysm wall enhancement increases the instability of IA (5); it is related to an increased level of atherogenic proteins and a decreased level of anti-atherosclerotic proteins, and atherosclerosis can be detected when these enhanced arterial walls are observed *in vitro* (6, 7). In addition, inflammation-related atherosclerotic changes and neovascularization of the aneurysm wall have been found in larger unruptured IAs (4), and increased lipid infiltration was observed in the ruptured cerebral aneurysm wall (8). In summary, atherosclerosis-related intracranial aneurysms (AS-related IA) are more unstable and require further care in clinical practice.

**Figure 1:**
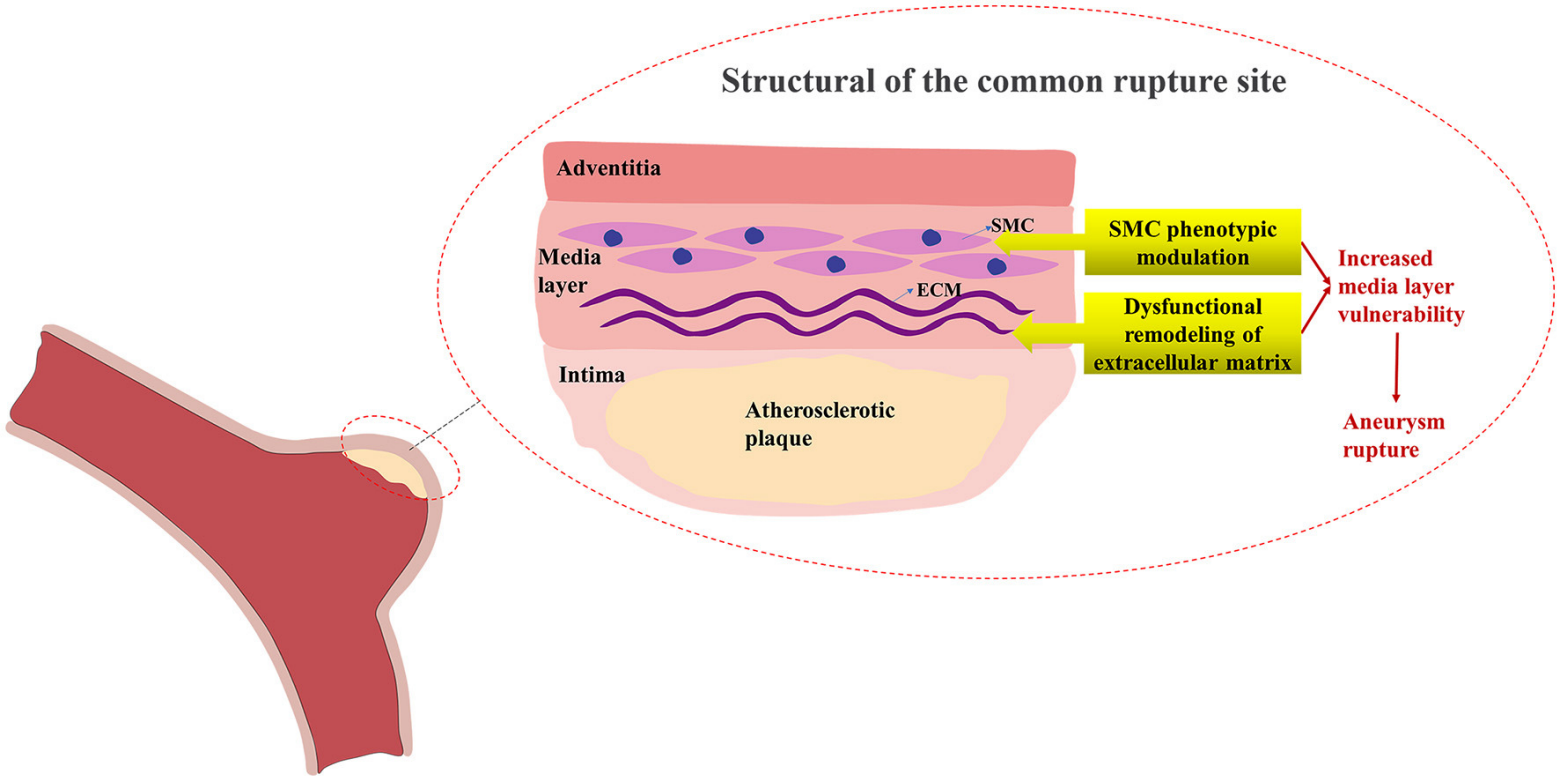
Structural alterations of common rupture sites in AS-related IA.

Although atherosclerosis is considered one of the risk factors for IA, the pathogenic mechanism of the complication of IA and AS remains unknown but may be connected to inflammation, smooth muscle cell (SMC) proliferation, and macrophage phagocytosis (9). Currently, histological studies have demonstrated that the SMC phenotype, lipoprotein buildup, and production of foam cells in intracranial aneurysm walls are similar to the alterations in atherosclerotic artery walls (10–12), indicating that there may be some common mechanisms leading to the occurrence of these two diseases and triggering the onset of AS-related IA. The increased rupture risk of AS-related IA may be mainly caused by atherosclerosis-induced phenotypic modulation of SMC in the aneurysm media layer (13). The adventitia of intracranial aneurysms comprises collagen fibers, encasing the media layer primarily composed of the SMC and extracellular matrix (ECM), while the intima is the invasive site of atherosclerotic plaques. When atherosclerosis occurs on the wall of intracranial aneurysms, the SMC of the media layer transforms to the matrix remodeling phenotype, resulting in ECM dysfunctional remodeling and the destruction of elastic fibers (14). These pathological alterations reduce the stability of the media layer of the aneurysm wall, increasing the risk of AS-related IA rupture (Figure 1).

With the gradual revelation of the close association between IA and AS, there is still a lack of effective treatments, and new strategies are urgently required to prevent corresponding adverse prognoses. This study aimed to identify the transcriptome signature of AS-related IA. We retrieved differentially expressed genes (DEGs) of IA and AS from the Gene Expression Omnibus (GEO) database and used integrative bioinformatics tools to uncover functional pathways, potential hub genes, and transcription factors. Our findings are expected to shed new insights into the pathogenic mechanisms and treatments of IAs complicated with atherosclerosis.

## 2. Materials and methods

### 2.1. Data source

GEO (http://www.ncbi.nlm.nih.gov/geo/) is a vast online database containing various high-throughput sequencing data types. We downloaded sequencing datasets of IA (GSE75436) and atherosclerotic vascular specimens (GSE100927) from the GEO database. GSE75436 comprised 15 IA wall tissues and 15 matched control superficial temporal artery walls. GSE100927 comprised 69 atherosclerotic samples and 35 control arteries without atherosclerosis.

### 2.2. Differential expression analysis

First, the acquired data were normalized, background adjusted, and log2 transformed; the probes without gene annotation were removed, and the values of duplicate probes were averaged. We performed differential gene expression analysis on GSE75436 and GSE100927 using the “limma” R package (https:/www.bioconductor.org/packages/3.5/bioc/html/limma.html) (15). DEGs were identified as genes with an adjusted *p*-value of <0.05 and a |logFC| of >1. Subsequently, we used Venny2.1 (http://bioinfogp.cnb.csic.es/tools/venny/index.html) to generate a Venn diagram of the intersection of the DEGs in these two datasets. Removing genes with opposite expression trends, we obtained the common DEGs (co-DEGs) in these two diseases.

### 2.3. Enrichment analyses of DEGs

Gene Ontology (GO) is a database that describes the related biological processes, molecular functions, and cellular components for gene collections. Kyoto Encyclopedia of Genes and Genomes (KEGG) is supported by a database containing functional annotation and gene pathway enrichment across multiple species. The co-DEGs were submitted to enrichment analyses using the clusterProfiler package (16), with an adjusted *p*-value of <0.05 serving as the screening criteria. The results were displayed using the Ggplot2 package (https://ggplot2.tidyverse.org).

### 2.4. Protein–protein interaction network construction and module analysis

The Search Tool for the Retrieval of Interacting Genes (STRING; http://string-db.org) is a database that can search for potential relationships between proteins (17) using the STRING database to construct a PPI network of co-DEGs with a combined score of >0.40 for meaningful interactions. To visualize the PPI network, we imported the results into Cytoscape (http://www.cytoscape.org) (18); subsequently, the MCODE plugin was used to find the potential meaningful gene modules in co-DEGs. Finally, we conducted an enrichment analysis on the most valuable gene modules.

### 2.5. Selection and analysis of candidate hub genes

Using the cytoHubba plugin in Cytoscape, we analyzed the entire PPI network. DEGs were analyzed in cytoHubba using 12 algorithms (MCC, DMNC, MNC, Degree, EPC, BottleNeck, EcCentricity, Closeness, Radiality, Betweenness, Stress, and ClusteringCoefficient), and each algorithm recorded the top 20 genes. Upset charts were constructed, calculating the frequency of gene registrations. Subsequently, we selected the genes reported six times or more as potential hub genes. GeneMANIA (http://www.genemania.org/) (19) is a dependable instrument for determining gene correlations. Candidate hub genes were imported into GeneMANIA for analysis.

### 2.6. Verification and analysis of hub genes

Two external datasets, GSE43292 (AS) and GSE122897 (IA), were used to verify the expression of candidate hub genes. GSE43292 included 32 carotid atherosclerotic plaques paired with 32 distant macroscopic control tissue samples; GSE122897 included 44 IA samples and 16 control intracranial cortical arterials. Data between groups were compared using the mean *t*-test, with a *p*-value of <0.05 as the standard for significant differences. The candidate genes that passed the verification were considered hub genes, and enrichment analysis was performed on them.

### 2.7. Prediction and verification of transcription factors

ChEA3 (https://maayanlab.cloud/chea3/) is an internet transcription factor (TF) enrichment analysis instrument that can predict the regulatory relationship between TFs and their corresponding target genes (20). The ENCODE TF target library contains ChIP-seq experiments from humans and mice. We imported hub genes into the ChEA3 database, set library = “ENCODE”, and predicted the top 10 TFs corresponding to them. Thereafter, we verified the expression levels of these TFs in GSE75436 and GSE100927.

## 3. Results

### 3.1. Differential expression analysis

**Figure 2:**
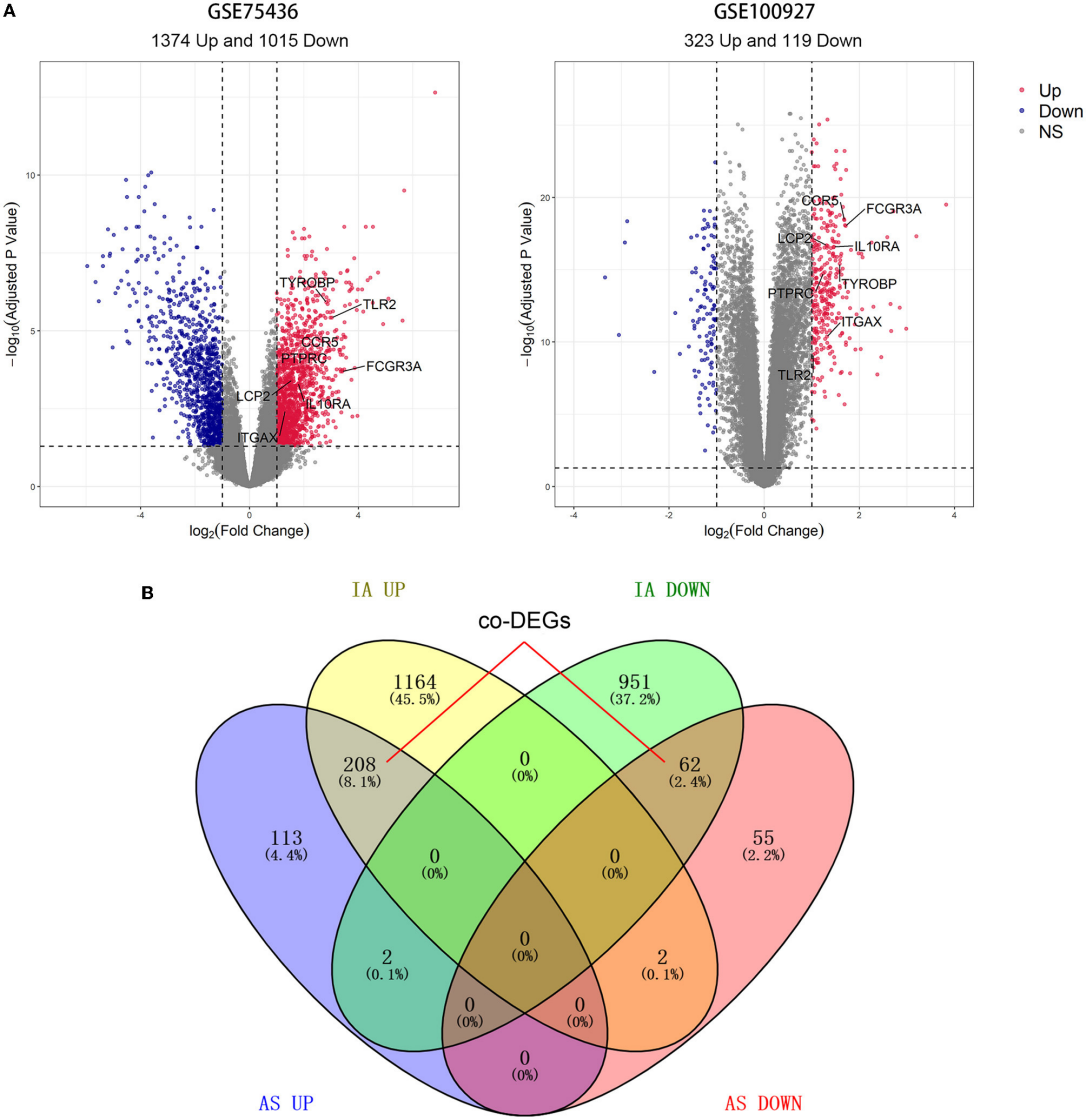
Results of differential expression analysis and intersection Venn diagram. **(A)** The volcano map of GSE75436 and GSE100927. Bright red color indicates upregulated genes; blue color indicates downregulated ones. **(B)** From the DEGs of the two datasets, 270 co-DEGs were selected.

Differential expression analysis revealed that GSE75436 included 2,389 DEGs comprising 1,374 upregulated DEGs and 1,015 downregulated DEGs; GSE100927 contained 442 DEGs consisting of 323 upregulated DEGs and 119 downregulated DEGs (Figure 2A). DEGs with the same expression trend in IA and AS were considered co-DEGs. Taking the intersection of DEGs and removing genes that had opposite expression trends in the two diseases, we obtained 208 upregulated co-DEGs and 62 downregulated co-DEGs (Figure 2B).

### 3.2. Enrichment analyses of DEGs

**Figure 3:**
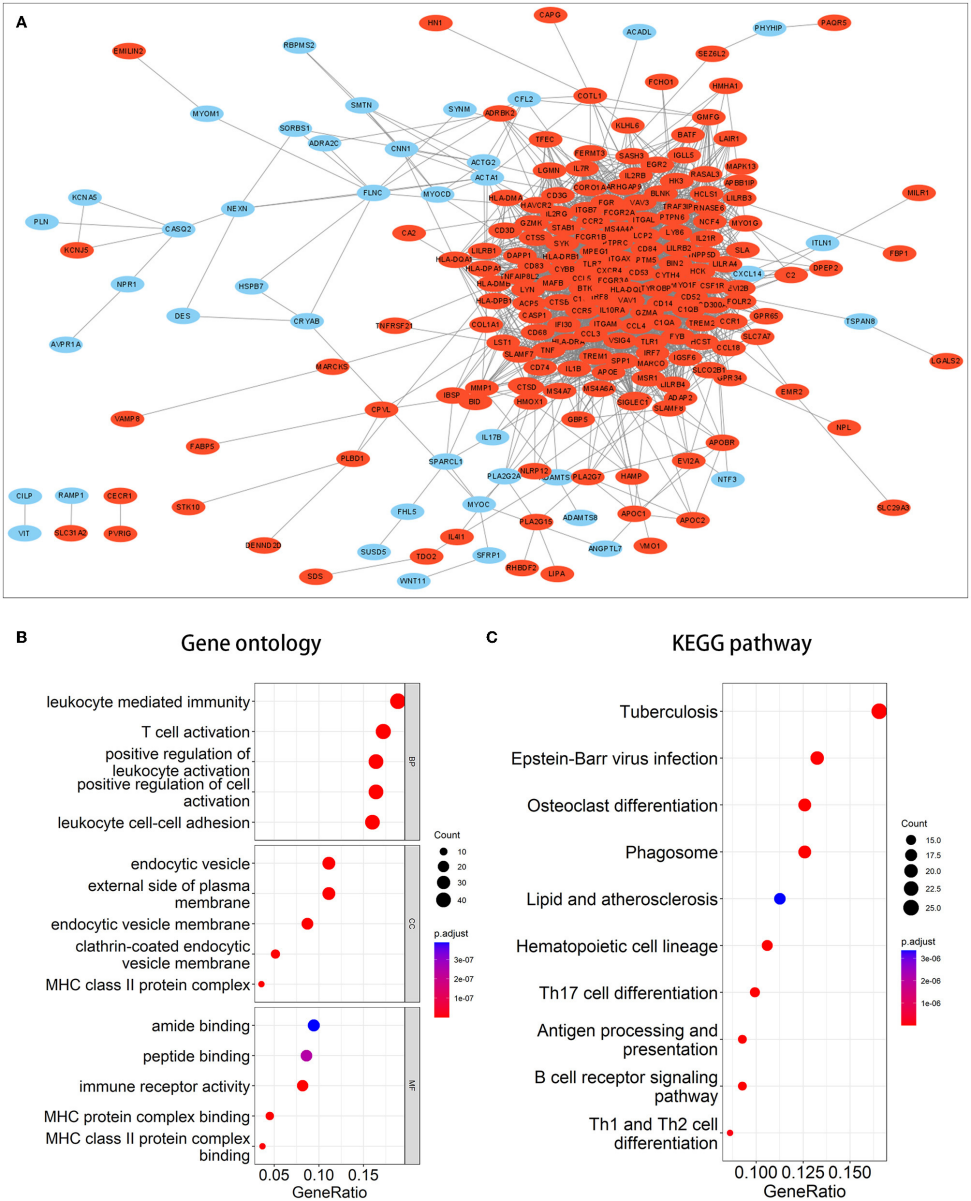
PPI network and enrichment analysis of common DEGs. **(A)** PPI network of common DEGs. Red signifies upregulated genes, while blue denotes downregulated genes. **(B, C)** The outcomes of GO and KEGG pathway enrichment analyses. It was deemed significant if the adjusted *P*-value of <0.05.

We performed enrichment analysis on 270 co-DEGs to explore their potential biological functions and pathways. According to GO analysis, these genes were primarily enriched in leukocyte-mediated immunity, leukocyte cell-cell adhesion, immune receptor activity, and endocytic vesicle, which were related to leukocyte immunity and cell phagocytosis (Figure 3B). Meanwhile, co-DEGs were substantially related to the phagosome, lipid, and atherosclerosis, and the B-cell receptor signaling pathway, as determined by KEGG analysis (Figure 3C). These results provide additional evidence that atherosclerosis induces the development of IA and reflect that immune response, phagocytosis, lipid accumulation, and other factors are implicated in the onset and progression of AS-related IA.

### 3.3. Construction and module analysis of the PPI network

**Figure 4:**
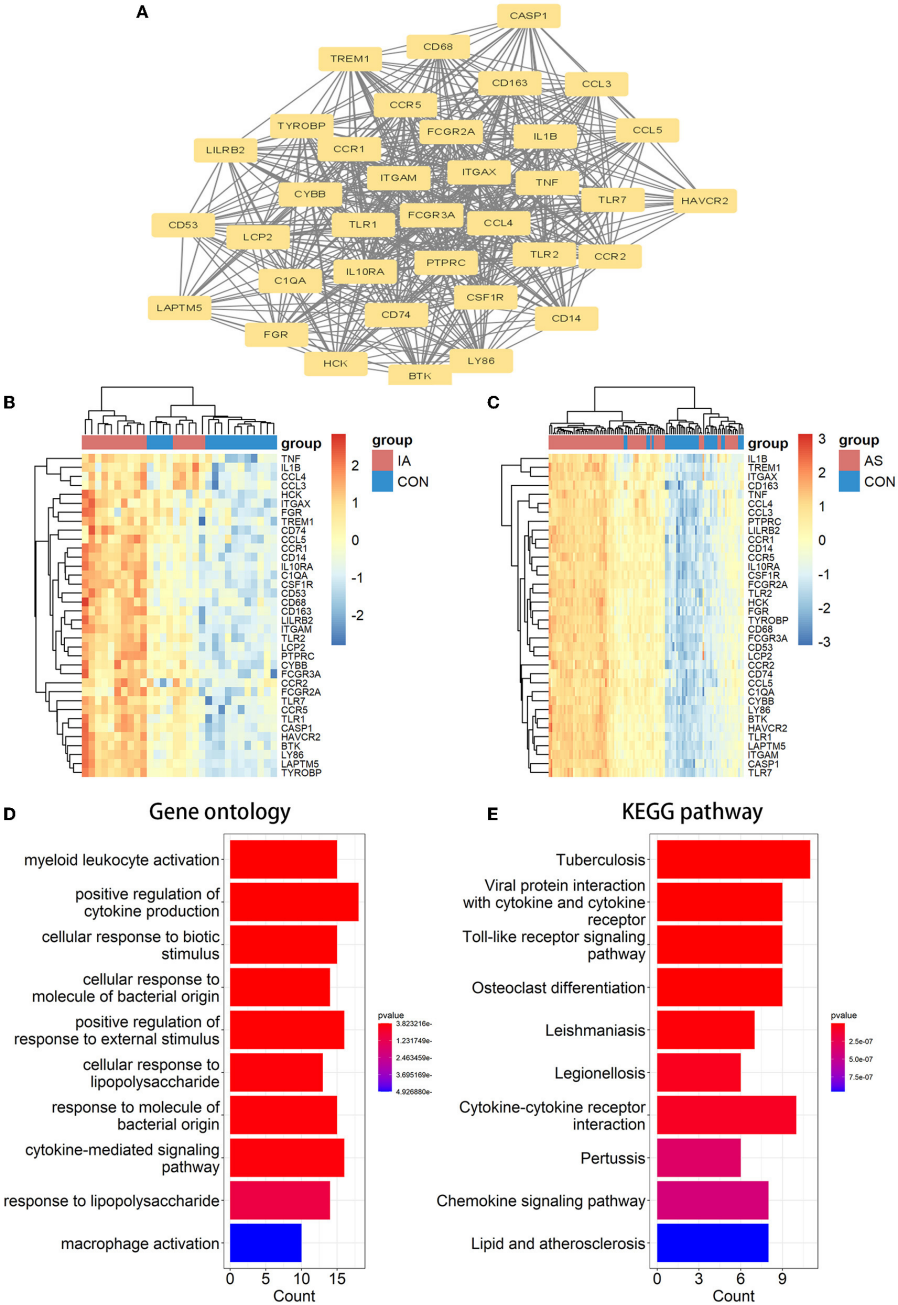
MCODE-identified genes module and corresponding enrichment analysis results. **(A)** One essential module of gene clustering. **(B, C)** Distribution of gene expression levels in the module. **(D, E)** The genes module underwent GO and KEGG analysis. It was deemed significant if the adjusted *P*-value < 0.05.

A PPI network of co-DEGs with 227 nodes and 2,379 interaction pairings was established (Figure 3A). Using the MCODE plugin (set *K*-core = 2, degree cutoff = 2, max depth = 100, and node score cutoff = 0.2), we analyzed this network and identified the most significant gene module (score = 28.857, 36 genes, and 505 interaction pairs) (Figure 4A). Interestingly, all genes in this module are upregulated co-DEGs, and heatmaps display their expression levels in GSE75436 (Figure 4B) and GSE100927 (Figure 4C). Moreover, we performed an enrichment analysis on this gene module. GO analysis revealed that these genes were predominantly engaged in immunologic inflammation and cell response regulation (Figure 4D), and KEGG analysis revealed their involvement in a toll-like receptor signaling pathway, a chemokine signaling route, lipid and atherosclerosis, and other pathways (Figure 4E).

### 3.4. Selection and analysis of candidate hub genes

**Figure 5:**
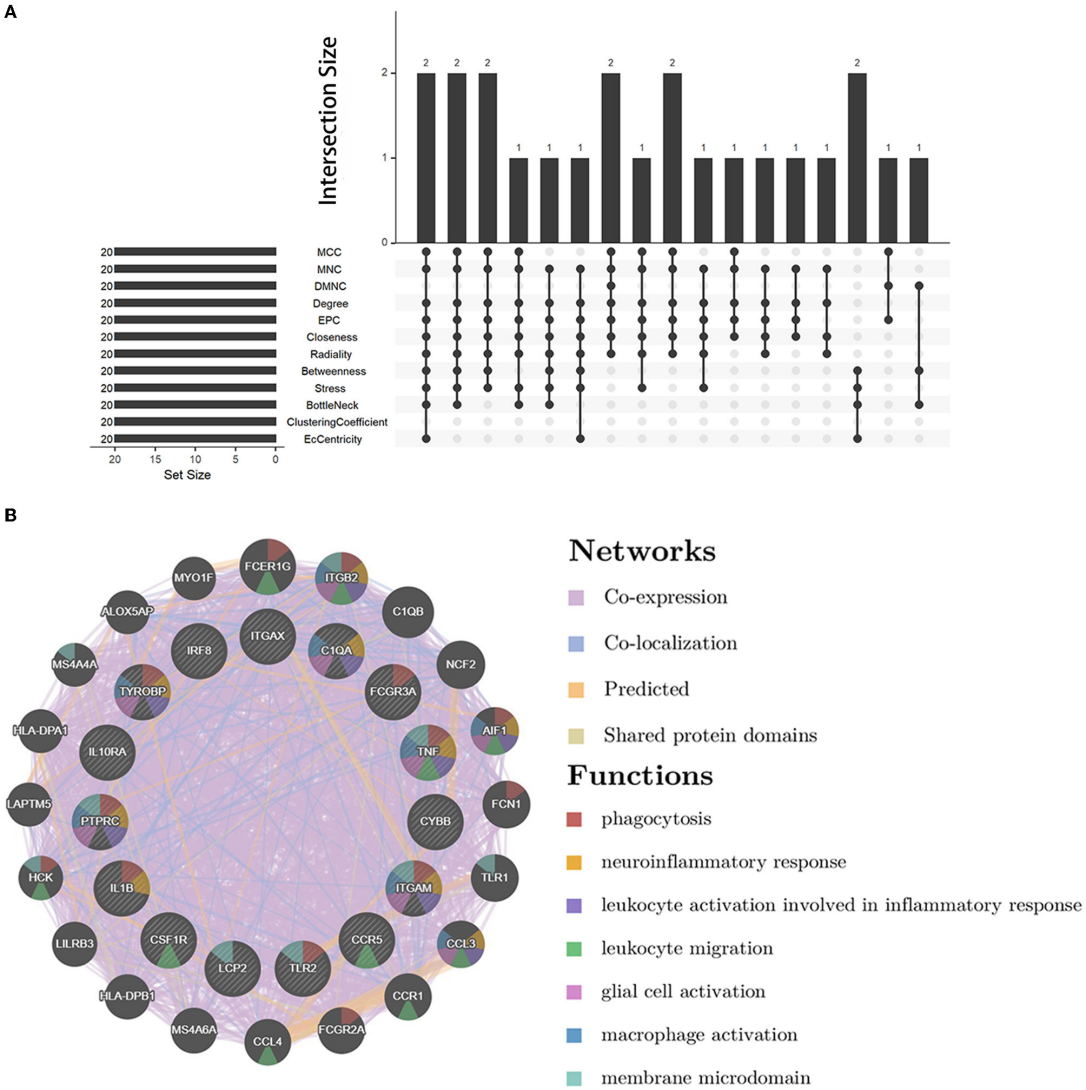
Candidate hub gene co-expression network and upset plot. **(A)** The intersection of the top 20 genes within 12 methods of the cytoHubba module. **(B)** GeneMANIA was used to analyze candidate hub genes and associated co-expression genes.

We analyzed the PPI network of co-DEGs using 12 algorithms in the cytoHubba plugin of Cytoscape software, and each algorithm obtained the top 20 candidate hub genes. According to the upset plot (Figure 5A), genes simultaneously selected by six or more algorithms were considered candidate hub genes, namely, PTPRC, TNF, ITGAM, TYROBP, IL1B, CSF1R, FCGR3A, IRF8, LCP2, TLR2, CYBB, CCR5, ITGAX, IL10RA, and C1QA, which were all upregulated genes. Based on the GeneMANIA database, we created gene co-expression networks and demonstrated their associated functions. These genes exhibited a complicated PPI network with 88.81% co-expression, 7.24% co-localization, 2.45% prediction, and 1.51% shared protein domains (Figure 5B).

### 3.5. Verification and analysis of hub genes

**Figure 6:**
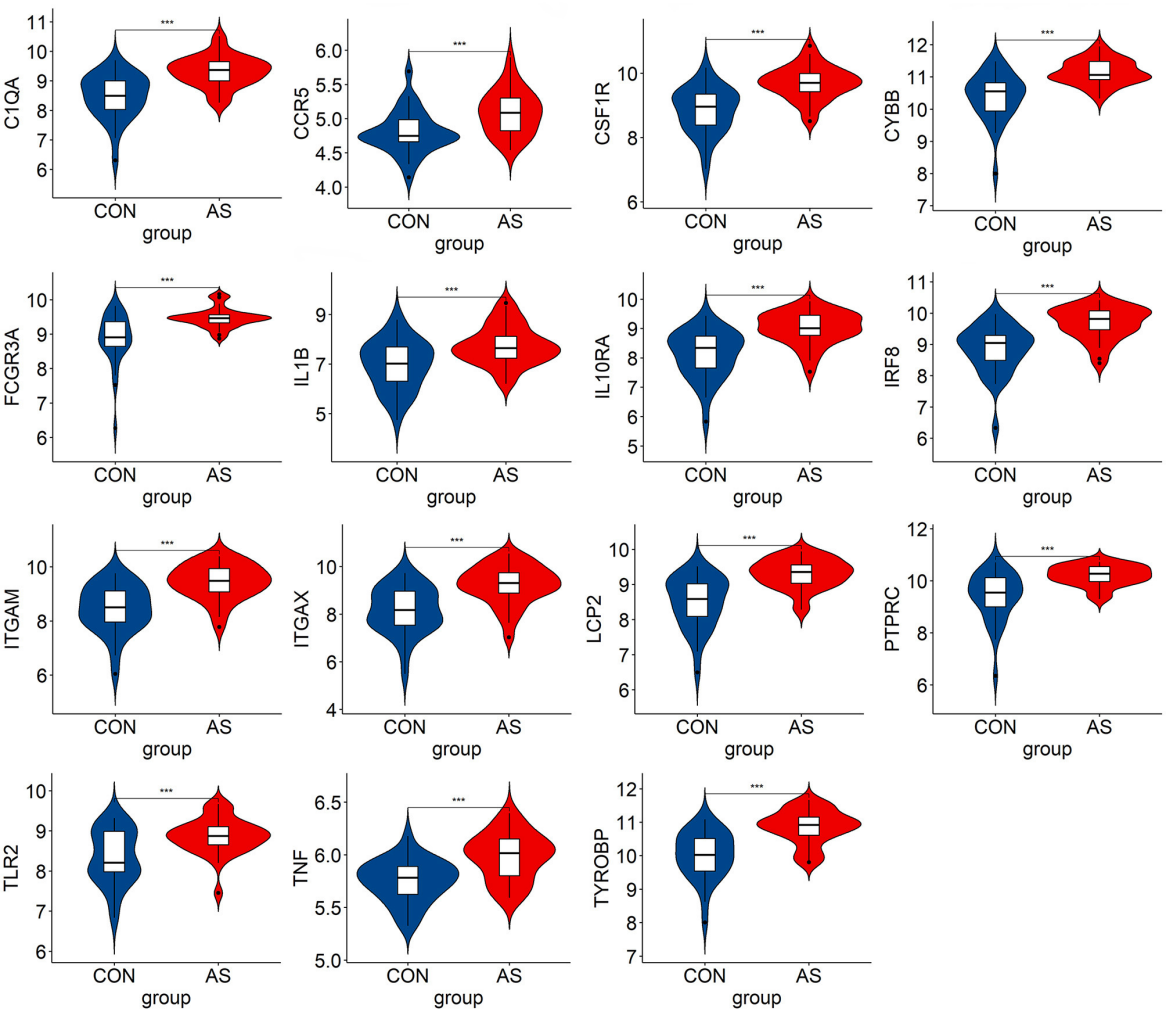
The hub genes expression level in GSE43292. The mean *t*-test is used for the comparison of the two datasets. Statistical significance was determined when the *P*-value < 0.05. AS, atherosclerotic plaque; CON, control artery samples. ***p < 0.001.

**Figure 7:**
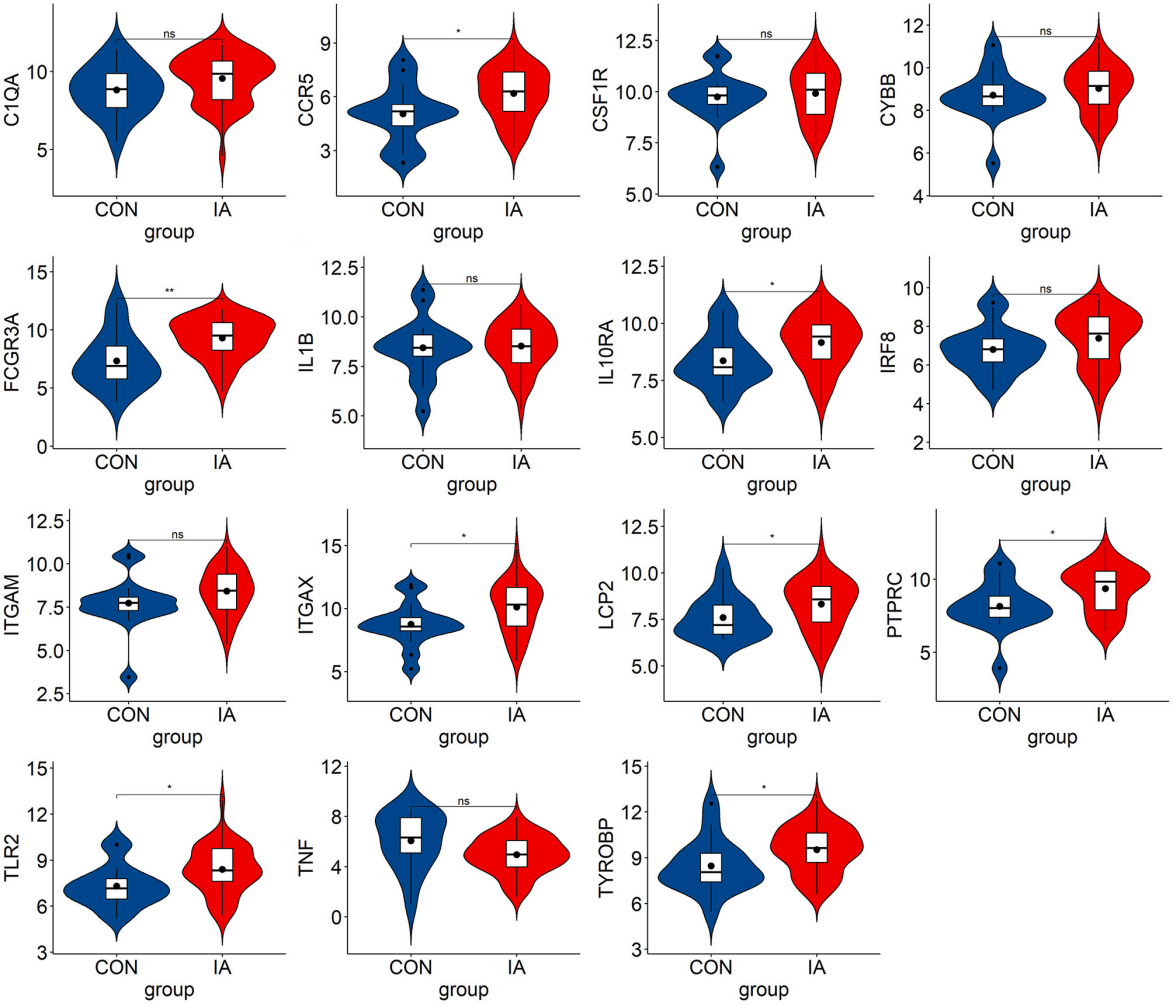
The hub genes expression level in GSE122897. The mean *t*-test is used for the comparison of the two datasets. Statistical significance was determined when the *P*-value < 0.05. IA, intracranial aneurysm samples; CON, control intracranial cortical arterials. *p < 0.05; **p < 0.01. ns, *p* > 0.05.

**Figure 8:**
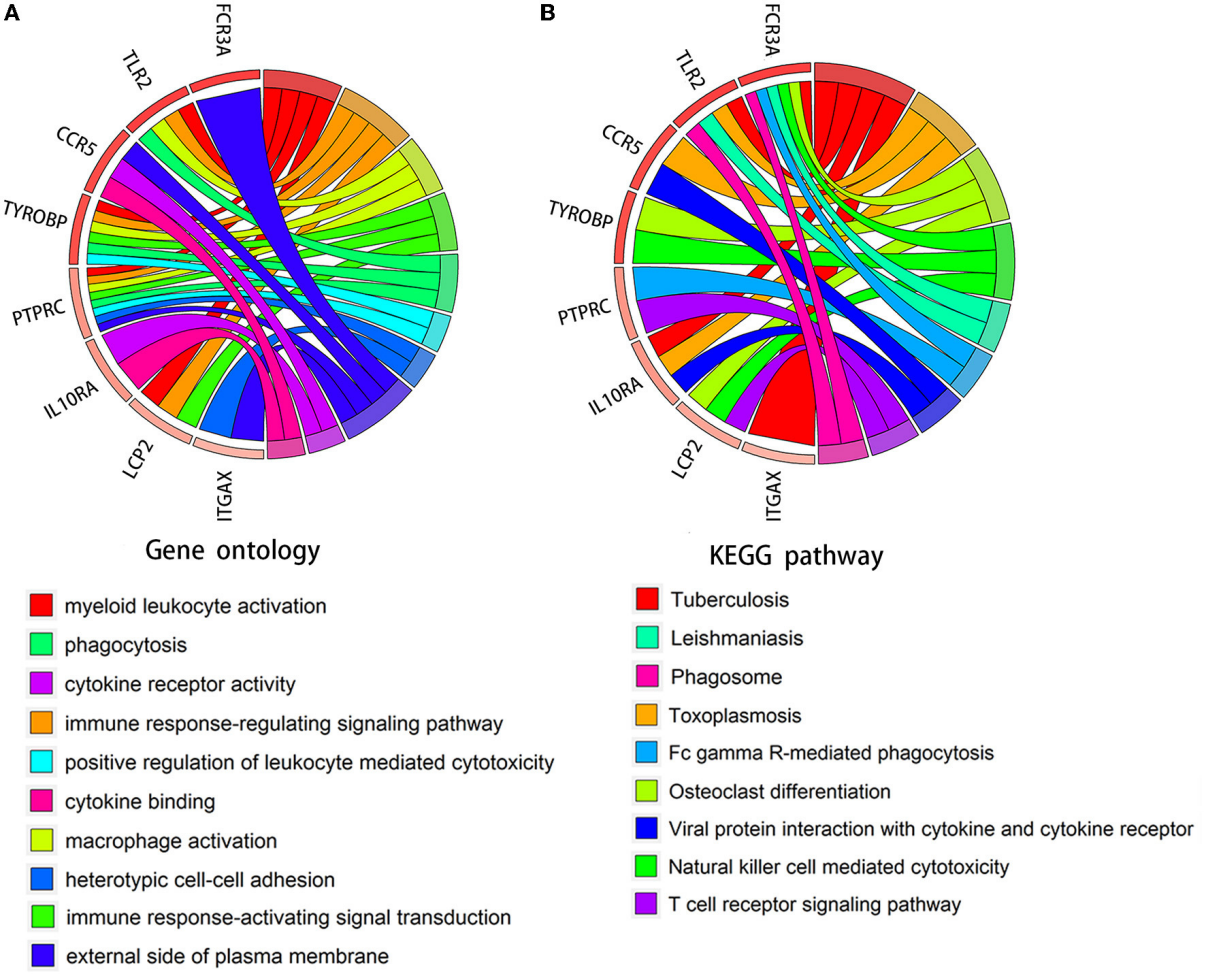
Chord diagram for enrichment analysis of hub genes. **(A, B)** GO and KEGG analysis of hub genes. The circles on the right and left, respectively, indicate pathways and the corresponding hub genes.

We verified the expression of 15 candidate hub genes with GSE43292 (AS) and GSE122897 (IA). All candidate hub genes were significantly upregulated in GSE43292 (Figure 6). While eight genes were remarkably upregulated in GSE122897, and the expression of candidate hub genes showed an overall upward trend in IA, except for IL1B and TNF (Figure 7). Eight genes were confirmed as hub genes of AS-related IA, namely, CCR5, FCGR3A, IL10RA, ITGAX, LCP2, PTPRC, TLR2, and TYROBP. Supported by the GeneCards database (https://www.genecards.org/), Table 1 displays their real names and associated functions. Interestingly, each hub gene corresponds to the MCODE algorithm's most significant gene module. Following the enrichment analysis of these genes, GO analysis demonstrated that they are mainly involved in the immune response, macrophage phagocytic function, and cytotoxicity (Figure 8A). Subsequently, their involvement in natural killer cell-mediated cytotoxicity, FcR-mediated phagocytosis, and the T cell receptor (TCR) signaling pathway were revealed by KEGG analysis (Figure 8B).

**Table 1:** Specifics and functions of hub genes.

**No**.	**Gene symbol**	**Full name**	**Function**
1	*PTPRC*	Protein Tyrosine Phosphatase Receptor Type C	PTPRC acts as a leukocyte antigen to regulate T- and B-cell immune signaling.
2	*TYROBP*	Transmembrane Immune Signaling Adaptor *TYROBP*	TYROBP triggers the mobilization of intracellular calcium ions, activates transcription factors such as *NF-κB*, and promotes the occurrence of cellular inflammatory responses.
3	*FCGR3A*	Fc Gamma Receptor IIIa	*FCGR3A* encodes the receptor for the Fc portion of immunoglobulin G and is involved in antibody-dependent biological processes such as mediating cytotoxicity.
4	*LCP2*	Lymphocyte Cytosolic Protein 2	LCP2 participates in the protein tyrosine kinase pathway activated by the T cell receptor, regulates helper T cell function, and promotes the activation of serine phosphorylation.
5	*TLR2*	Toll-Like Receptor 2	TLR2 acts on MYD88 to activate NF-κB, activates inflammatory response and cytokine secretion, promotes apoptosis, and affects the lipid portion of lipoproteins.
6	*CCR5*	C-C Motif Chemokine Receptor 5	As a receptor of inflammatory CC-chemokines, CCR5 can increase the intracellular calcium ion level and transduce signals and can affect the inflammatory immune process by participating in the migration of T lymphocytes to the site of action.
7	*ITGAX*	Integrin Subunit Alpha X	*ITGAX* encoded integrin α-X chain protein is involved in constituting leukocyte-specific integrins, mediates cell–cell interactions in inflammation, and plays a vital role in monocyte adhesion and chemotaxis.
8	*IL10RA*	Interleukin 10 Receptor Subunit Alpha	IL10RA can mediate IL-10 to transmit immunosuppressive signals and reduce the synthesis of pro-inflammatory cytokines.

### 3.6. Exploration and authentication of TFs

**Figure 9:**
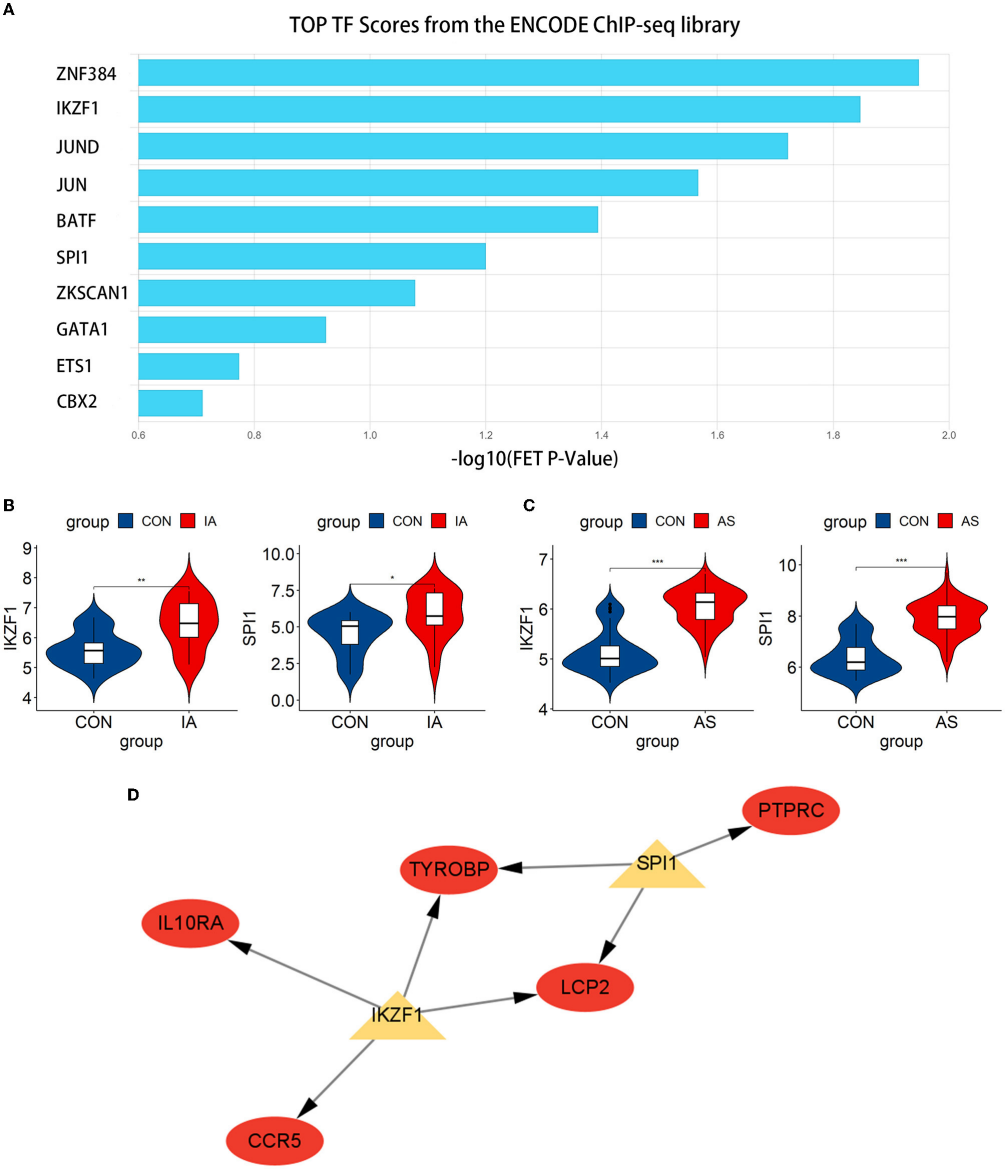
Exploration and verification of TFs and their regulatory networks. **(A)** Top 10 related TFs predicted using the ChEA3 database. **(B, C)** TFs expression level in GSE75436 and GSE100927. The mean *t*-test is used for the comparison of the two datasets. Statistical significance was determined when the *P*-value < 0.05. IA, intracranial aneurysm wall tissue; AS, atherosclerotic samples; CON, control arteries. *p < 0.05; **p < 0.01; ***p < 0.001. **(D)** The regulatory network of TFs. The hub genes were highlighted in red, whereas TFs were highlighted in yellow.

We predicted the top 10 TFs that may regulate hub gene expression using the ChEA3 database (Figure 9A). Thereafter, we verified the expression of these TFs in datasets. In total, two TFs, IKZF1 and SPI1, were significantly upregulated in GSE75436 (Figure 9B) and GSE100927 (Figure 9C). Subsequently, a network diagram of the TFs and hub genes was constructed. A total of five hub genes (CCR5, IL10RA, LCP2, TYROBP, and PTPRC) were coregulated by these two TFs (Figure 9D).

## 4. Discussion

This study determined new targets for preventing and treating AS-related IA, revealing the potential biological mechanism in the pathogenesis. A total of 270 co-DEGs were identified, and then eight hub genes were selected and verified, namely, CCR5, FCGR3A, IL10RA, ITGAX, LCP2, PTPRC, TLR2, and TYROBP. After the enrichment analysis of hub genes, we found that these genes were principally enriched in cytotoxicity, phagocytosis, and TCR signaling pathways. SPI1 and IKZF1, two transcription factors, were discovered to be significant for the development of this disease, and they jointly regulate five hub genes, namely, CCR5, IL10RA, LCP2, TYROBP, and PTPRC.

The enrichment analysis of hub genes showed that NK cell-mediated cytotoxicity, FcγR-mediated phagocytosis, and the TCR signaling pathway might play considerable roles in the pathogenesis of AS-related IA. The atherosclerotic tissue is rich in NK cells that express many biomarkers, such as IFN-γ. NK cell activation may play a significant role in the exacerbation of AS (21), suggesting that the increased cytotoxicity mediated by NK cells may contribute to the development of AS. Moreover, the migration ability of NK cells in the peripheral blood of patients with IA is also enhanced (22). NK cells may aggregate and activate in the aneurysm wall during the pathogenesis, which mediates cytotoxicity to promote disease progression. In addition, phagocytosis appears to be activated during pathogenesis. FcγR is a receptor for the Fc portion of IgG, which activates the mitogen-activated protein kinase signaling pathway by mediating low-density lipoprotein immune complexes (LDL-ICs), thereby activating macrophages (23, 24). Subsequently, macrophage infiltration and its polarization toward the M1 phenotype increase the risk of IA pathogenesis and rupture (25). Inflammatory macrophages in the arterial wall can uptake LDL-ICs through FcγRI and transform them into foam cells (26), forming atherosclerotic plaques. FcγR may activate inflammatory macrophages and promote their phagocytosis to induce the aggregation of foam cells, leading to the development of atherosclerotic plaque in the aneurysm wall, inducing the deterioration of IA. The activation of the TCR signaling pathway will lead to the cascade reaction of the PKCθ-IKK-NFκB pathway (27), stimulating the NFκB-mediated inflammatory response and participating in the pathology process of AS-related IA.

As a receptor of inflammatory CC-chemokines, CCR5 can increase intracellular calcium ion levels to transduce signals. CCR5 transports blood monocytes to atherosclerotic plaques to promote disease progression (28). Cipriani et al. treated AS model mice with a CCR5 antagonist, which resulted in a 70% reduction in plaque volume and a 50% attenuation of monocyte/macrophage infiltration (29). T cells in the wall of IA express high levels of the chemokine receptor CCR5 (30). We speculate that CCR5 may promote disease progression by participating in the chemotactic process of inflammatory macrophages in the aneurysm wall. FCGR3A, also known as CD16, is involved in mediating cytotoxicity. Previous studies have found more CD16+ intermediate monocytes in patients with IA (31). Decreased CD16 monocyte subsets are also associated with a decrease in subclinical AS (32). Combined with our study, FCGR3A may induce disease development by promoting the cytotoxic effect of monocytes and macrophages. The protein encoded by IL10RA is a receptor for interleukin 10 (IL10); It can mediate immunosuppressive signals and reduce inflammatory responses. Patients with IA exhibit a decrease in IL-10, suggesting that the low IL-10 level *in vivo* may be associated with the development of IA (33). Moreover, the IL10RA was highly expressed in AS (34), indicating that the IL10RA-mediated inhibition of inflammation is similarly active in AS. Therefore, IL10RA may play a protective role in AS-related IAs by mediating the anti-inflammatory effect of IL-10, and activating the expression of IL10RA can effectively prevent the occurrence of AS-related IA. ITGAX, also known as CD11c, can mediate cell–cell interactions in inflammation, monocyte adhesion, and chemotaxis. The decrease in CD11c + cells can reduce the progression of abdominal aortic aneurysms (AAA) (35). According to our results, ITGAX may have a similar function in IA. Simultaneously, the high expression of ITGAX is a prominent feature of unstable carotid atherosclerotic plaques. CD11c + macrophages gather in vulnerable plaques, resulting in the deterioration of AS (36). The effect of ITGAX on AS-related IA may depend on affecting the adhesion and chemotaxis of monocytes.

PTPRC, also known as CD45, encodes a leukocyte antigen that regulates the immune response of T and B cells. PTPRC is involved in the progression of AS as a regulatory T cell-related gene (37). Hosaka et al. found the infiltration of CD45+ cells in IA walls (38), reflecting the involvement of PTPRC in the pathogenesis of IA. PTPRC may promote the inflammatory response of AS-related IA by regulating immune lymphocytes. TLR2 activates the inflammatory response and cytokine secretion by activating the TLR2-Myd88-NF-κB pathway and can also activate immune cells to promote apoptosis. Multiple studies have demonstrated that the TLR2-Myd88-NF-κB pathway is activated in IA and AS (39, 40). TLR2 may promote the pathogenesis of AS-related IA mainly by activating the inflammatory response mediated by the TLR2-Myd88-NF-κB pathway and apoptosis. TYROBP, also known as DAP12, encodes a protein that can activate TFs, such as NF-κB, and promote cellular inflammatory responses. Previous studies have found that the expression level of TYROBP is significantly upregulated in the atherosclerotic tissue and AAA, and TYROBP promotes the pathogenesis of AAA through the activation of the NK cell-mediated cytotoxicity pathway (41, 42). Combined with our results, TYROBP may also have such a role in the progression of AS-related IA, which may lead to disease by activating NF-κB and affecting NK cell-mediated cytotoxicity. The association between LCP2 and AS-related IA is still unclear, and the underlying mechanism requires additional investigation.

Subsequently, we predicted and verified the TFs of hub genes. Among the top 10 TFs predicted from the ChEA3 database, IKZF1 and SPI1 passed the verification, and they jointly regulated five hub genes, namely, CCR5, IL10RA, LCP2, TYROBP, and PTPRC. IKZF1 is considered a transcriptional regulator of hematopoietic differentiation and participates in the development of lymphocytes, B cells, and T cells (43). IKZF1 can promote the production of the inflammatory cytokine INF-γ by regulating the balance of Th1/Th2 (44). Increased IFN-γ levels can be observed in patients with IA, especially when the aneurysm ruptures (33). Moreover, the severity of AS is associated with genetic polymorphisms in the arterial IFN-γ gene (45). IFN-γ affects immune cells, endothelial cells, and SMCs (46, 47), leading to the progression of AS and may play a similar role in the pathogenesis of AS-related IA. IKZF1 is involved in regulating the expression of CCR5, IL10RA, LCP2, and TYROBP and can also promote the production of INF-γ to affect the pathogenesis process. IKZF1 may become a new therapeutic target. Another TF, SPI1, regulates LCP2, PTPRC, and TYROBP. SPI1 was significantly upregulated in aortic atherosclerotic plaques in Tibetan minipigs (48). In addition, SPI1 was identified as a significant regulator in peripheral blood samples of patients with IA (49), which also corresponds to the results obtained in our study. Due to the lack of relevant studies, the relationship between SPI1 and AS-related IA still needs to be explored.

Our findings contribute to elucidating the mechanism of the link between IA and AS. However, there are still some flaws in our study. First, the findings of our retrospective study need to be further confirmed with external data. Second, hub genes need to be further verified experimentally in *in vitro* models. Complementing these shortcomings is the focus of our future study.

## 5. Conclusion

We identified and verified eight hub genes and two TFs for AS-related IA, providing new study directions and therapeutic targets for this disease. Eight genes, namely, *CCR5, FCGR3A, IL10RA, ITGAX, LCP2, PTPRC, TLR2*, and *TYROBP*, are considered hub genes, and their pathway enrichment results focus on phagocytosis, NK cell-mediated cytotoxicity, and the TCR signaling pathway. Moreover, IKZF1 and SPI1 were identified as the TFs of hub genes and jointly involved in regulating the expression of five genes, namely, CCR5, IL10RA, LCP2, TYROBP, and PTPRC.

## Data availability statement

The original contributions presented in the study are included in the article/supplementary material, further inquiries can be directed to the corresponding author.

## Ethics statement

Ethical review and approval was not required for the study on human participants in accordance with the local legislation and institutional requirements. Written informed consent from the patients/participants or patients/participants' legal guardian/next of kin was not required to participate in this study in accordance with the national legislation and the institutional requirements.

## Author contributions

QZ completed the data analysis and the writing of the manuscript. HL critically revised the manuscript. MZ checked for omissions in the study and provided comments. FL and TL participated in the formulation of the draft study design. The final manuscript has been read and approved by all authors.
